# The Effect of Nasal Obstruction after Different Nasal Surgeries Using Acoustic Rhinometry and Nasal Obstruction Symptom Evaluation Scale 

**Published:** 2016-09

**Authors:** Erkan Kahraman, Yakup Cil, Armagan Incesulu

**Affiliations:** 1 Department of Otorhinolaryngology, Eskisehir Military Hospital, Eskisehir, Turkey;; 2 Department of Plastic, Reconstructive and Aesthetic Surgery, Etimesgut Military Hospital, Ankara, Turkey;; 3 Department of Otorhinolaryngology, Faculty of Medicine, Eskişehir Osmangazi University, Eskisehir, Turkey

**Keywords:** Nasal obstruction, nasal surgery, acoustic rhinometry, NOSE scale

## Abstract

**BACKGROUND:**

The efficiency of nasal surgeries can be determined by objective or subjective methods. We have assessed the effect of nasal obstruction after different nasal surgeries using Acoustic Rhinometry (AR) and Nasal Obstruction Symptom Evaluation (NOSE) Scale.

**METHODS:**

Between May 2011 and May 2012, 40 young adult patients and 10 healthy volunteers as control group who referred to Otorhinolaryngology Clinic in Eskisehir Military Hospital due to nasal obstruction were enrolled. Depending on operation, patients were divided into four equal groups. Group 1: Septoplasty, Group 2: Septoplasty with sprader graft, Group 3: Septorinoplasty and Group 4: Septorhinoplasty with spreader graft. The patients completed NOSE scale, 1 week before and 1 month after the surgery and AR measurements.

**RESULTS:**

There were a significant improvement in mean NOSE scores of patients and statistical difference was found between pre and post-operational values for each group. There was a statistically significant change of the mean minimal cross section areas (MCA) of the deviated side of nasal passages measured by AR between pre and postoperative period.

**CONCLUSION:**

In patients with nasal obstruction, functional nasal surgeries which were performed after appropriate medical examination and with right operation methods had a positive impact on quality of life and patient satisfaction. We observed that nasal findings were correlated with NOSE scores and MCA values. So, we suggest that NOSE scale and AR to be used for evaluation of the efficiency of functional nasal surgeries.

## INTRODUCTION

Nasal obstruction is one of the most common problems in otolaryngology practice. Nasal obstruction can be caused by several factors such as deviation of nasal septum, nasal valve collapse, turbinate hypertrophy and nasal polyposis.^1^ Among them, septum deviation is the main etiologic factor and more than half of the population have this problem.^2^^,^^3^ The main purpose of functional nasal surgery is to improve nasal breathing function. For this reason, the nasal septum deformity should be corrected first. Septoplasty is an effective surgical treatment for correction of septal deviation. For septonasal deformities, septorhinoplasty is frequently performed which aims to correct functional condition and external deformities of the nose. The internal nasal valves may contribute to half of total airway resistance and deformities of this area can cause important nasal obstruction.^4^ Several methods including spreader graft, batten grafts and flare sutures are used to support nasal valve area and to avoid nasal obstruction.^5^

As nasal obstruction is subjective and difficult to evaluate by clinical examination and also this subjective feeling of nasal obstruction can be deceptive. There are 2 methods to evaluate results after functional nasal surgery: objective and subjective measures. Surgical achievement can hardly be assessed with subjective measurements. That is why objective assessment of nasal function should be used. Nasal Obstruction Symptom Evaluation Scale (NOSE scale) is used as a subjective method. NOSE scale is a disease-specific quality of life instrument for use in nasal obstruction and it was used in several studies.^6^

NOSE scale is a short, valid and reliable method and also seems as a valuable subjective instrument for evaluating functional nasal surgery. Currently one of the most commonly used objective methods is acoustic rhinometry (AR). AR is based on analysis of sound waves reflected from the nasal cavity. It is a noninvasive, simple and quick technique. AR is used for the evaluation of patients undergoing septoplasty or other nasal surgeries, and it can provide objective information for the surgeon. AR has been used in other nasal operations to determine surgical effects, such as in turbinoplasty^7^ and endoscopic sinus surgery.^8^

In this study, we aimed to evaluate of the increase in life quality of young adult patients among the ones who suffered from nasal obstruction and were subjected to 4 different nasal surgeries including septoplasty, septoplasty with spreader graft, septorhinoplasty and septorhinoplasty with spreader graft by using objective (AR) and subjective (NOSE scale) methods.

## MATERIALS AND METHODS

This study included 40 young adult patients who underwent four different nasal surgeries and 10 healthy volunteers as control group, at a time period of nearly 12 months between May 2011 and May 2012. Main complaints of the patients were nasal obstruction. All patients were informed about the study and informed consent was obtained in each case before surgery. Ethics committee approval was obtained from the Eskisehir Osmangazi University. Inclusion criteria were as follows: at least 18 years old, septal deviation consistent with nasal obstruction at least for 3 months, persistent symptoms after a 4-week trial of medical management (nasal steroid, antihistaminic and/or oral decongestants). 

Exclusion criteria were as follows: sinonasal malignancy, prior nasal surgery (such as septoplasty, septorhinoplasty, endoscopic sinus surgery, nasal valve surgery, turbinate surgery, etc.), sinonasal infections and inflammatory diseases, septal perforation, craniofacial syndrome, nasal trauma or fracture and adenoid hypertrophy. Comprehensive preoperative anamnesis was obtained from all patients. They underwent routine ear nose throat physical examination. The diagnosis was performed with anterior rhinoscopy and 0° rigid endoscope. The presence of valve problem was evaluated by modified Cottle test and endoscopic nasal examination.

Depending on the applied operation, patients were divided into four different groups, each of which had ten patients. Group 1: patients with a diagnosis of isolated septal deviation and so they underwent septoplasty, Group 2: patients with a diagnosis of septal deviation with nasal valve collapse and so they underwent septoplasty with spreader graft, Group 3: patients with a diagnosis of septonasal deformities and so they underwent septorinoplasty and Group 4: patients with a diagnosis of septonasal deformities with nasal valve collapse and so they underwent septorhinoplasty with spreader graft. For spreader graft; the upper lateral cartilages were carefully dissected from the septum of the submucoperichondrial layer, and the connections were separated. 

**Fig. 1:**
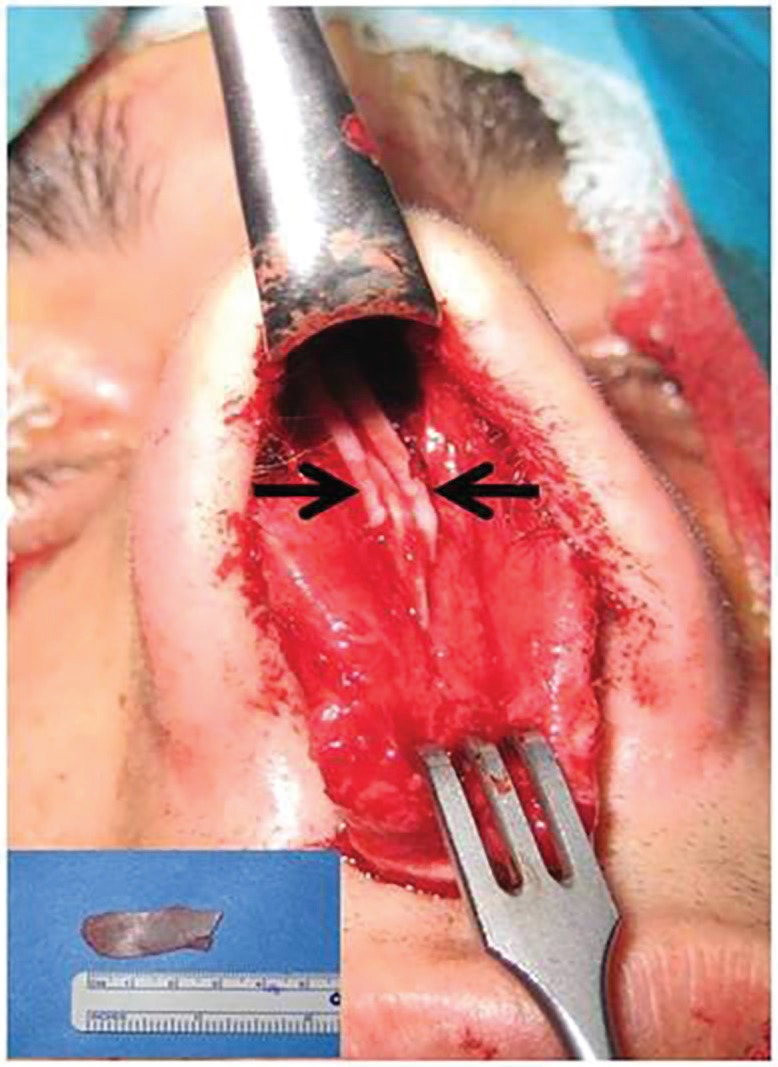
Spreader grafts obtained from nasal septal cartilage (small figure) was placed symmetrically and bilaterally along the dorsal edge of the remaining septal cartilage (black arrows

Septal cartilage was harvested, leaving a 10-mm L-shaped strut for nasal support. Two rectangular strips of cartilage were removed from the septum for use as spreader grafts and placed symmetrically and bilaterally along the dorsal edge of the remaining septal cartilage (Figure 1). All patients underwent surgery by the same surgeon using general anesthesia. Internal nasal splint or nasal packing was inserted at the end of the operation and removed 2 days after surgery. There were no serious complications in the postoperative period.

The patients were asked to complete NOSE scale, 1 week before and 1 month after the operation. The NOSE scale was used to assess disease specific quality of life and was scaled from 0 to 100, with higher scores meaning more severe nasal obstruction. Sum of the answers were multiplied by five to base the scale out of a possible score of a 100 for analysis. There are 5 questions in this scale. (i) Nose obstruction and stuffiness, (ii) Nose obstruction, (iii) Trouble breathing through my nose, (iv) Trouble sleeping and (v) Unable to get enough air through my nose during exercise or exertion (Table 1). 

**Table 1:** NOSE Scale (Nasal Obstruction Symptom Evaluation Scale

**NOSE Scale**	**Not a** **problem**	**Very mild** **problem**	**Moderate** **problem**	**Fairly bad** **problem**	**Severe** **problem**
Nose obstruction and stuffiness	0	1	2	3	4
Nose obstruction	0	1	2	3	4
Trouble breathing through my nose	0	1	2	3	4
Trouble sleeping	0	1	2	3	4
Unable to get enough air through my nose during exercise or exertion	0	1	2	3	4

The physician was blinded to the patient’s NOSE scores, before and after surgery. AR was performed 1 week before and 1 month after the operation by the same physician and using SRE 2000 Rhinometer (RhinoMetrics, Lynge, Denmark). Nasal cavities were cleaned by suction and prepared for AR. Measurements were performed with the patient in the sitting position after applying nasal decongestant (xylometazolin 0.01%, utilized three puffs for each nasal cavity) in a relatively quiet room at normal temperature (22-25^O^C) and humidity (50-60%). 

Patients were asked to hold their breath during the measurement. After 30 minute waiting period, three measurements were performed for both nasal passages separately. Minimal cross section area at the first 2 cm (MCA1), 2-5 cm (MCA2) of nasal cavity and nasal cavity volume at the first 2 cm (Vol1), 2-5 cm (Vol2) were recorded. An external nasal adapter was used, and each side of the nose was measured taking care to fit the nosepiece tightly to the nostril without distorting the anatomy.

The normality of the distribution was checked with Shapiro–Wilk’s test. The difference between pre and postoperative values between groups was evaluated by “One Way ANOVA” test. Statistical analyses were performed using SPSS software (Version 19.0 for Windows (SPSS Inc., Chicago, IL, USA). *p*<0.05 was accepted as the statistical significance level. For each group the differences between the values were evaluated by ​​“Paired samples T-test”. Pre and postoperative subjective symptoms were evaluated by “Repeated Measures ANOVA” test.

## RESULTS

This study population ranged from 19 to 23 years, with a mean of 21±2.1 years. All of the patients were male and admitted with nasal obstruction. Before operation, localization of deviated septum was determined for all of them. According to anterior rhinoscopy, nasal endoscopy and AR measurements, nasal obstruction was seen in right side for 17 patients and in left side for 23 patients. First, septum deviations were corrected with septoplasty for all patients, but no one had turbinate hypertrophy to block the nasal airway so turbinate surgery was not performed in any patient. They underwent 4 different nasal surgical treatments and assessed by both subjective (NOSE scale) and objective (acoustic rhinometry) methods. 

**Fig. 2:**
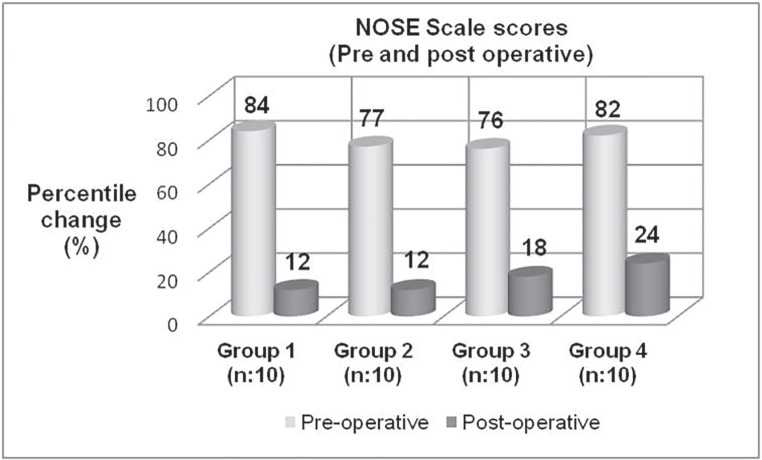
Pre and post-operative percentile change of quality of life between groups by NOSE scale (%). [Sums of the answers were multiplied by five to base the scale out of a possible score of a 100 for analysis

The normality of the distribution was checked with Shapiro–Wilk’s test and was found normal distribution (*p*>0.05). According to the NOSE scale results obtained before surgery (mean±standard deviation and percentile value); for group 1: (the patients who underwent septoplasty) 16.8±1.3 (84%); for group 2: (the patients who underwent septoplasty with spreader graft) 15.4±2.4 (77%); for group 3: (the patients who underwent septorinoplasty) 15.2±1.3 (76%); for group 4: (the patients who underwent septorinoplasty with spreader graft) 16.4±2.3 (82%). No statistically significant difference was found the preoperative NOSE scores between groups (*p*=0.500) (Table 2, Figure 2).

**Table 2:** Pre and post-operative NOSE Scale scores (mean±standard deviation and percentile value) (♦): Statistically significant (*p*<0.05).

**NOSE Scale**	**Pre-operative** **Mean±SD (%)**	**Post-operative** **Mean±SD (%)**	***p*** ** value**
Group 1 (n: 10)	16.8±1.3 (84)	2.4±0.5 (12)	*p*<0.05 (♦)
Group 2 (n: 10)	15.4±2.4 (77)	2.4±1.8 (12)	
Group 3 (n: 10)	15.2±1.3 (76)	3.6±1.1 (18)	
Group 4 (n: 10)	16.4±2.3 (82)	4.8±2.8 (24)	
*p* value	0.500	0.140	

According to the NOSE scale results obtained after surgery (mean±standard deviation and percentile value); for group 1: 2.4±0.5 (12%); for group 2: 2.4±1.8 (12%); for group 3: 3.6±1.1 (18%); for group 4: 4.8±2.8 (24%). No statistically significant difference was found the postoperative NOSE scores between groups (*p*=0,140) (Table 2, Figure 2). A significant improvement was noted on subjective symptoms of patients by Repeated Measures Anova test (p<0.05) and statistical difference was found between pre and post-operational values for each group by Paired samples T test (*p*<0.05).

Pre and postoperative results of AR measurements (MCA and nasal cavity volumes) were evaluated for deviated and non-deviated side of nasal passages for each patient. The changes in the nasal valve were best demonstrated by MCA1, and changes in the nasal septum and concha are best demonstrated by MCA2. Compared with mean pre and postoperative minimal cross-sectional areas of nasal cavity values (MCA1 and MCA2), there was a statistically significant increase in the mean postoperative MCA1 values (Group 1: 0.56±0.24 cm^2^, Group 2: 0.54±0.29 cm^2^, Group 3: 0.49±0.10 cm^2 ^and Group 4: 0.51±0.26 cm^2^) and MCA2 values (Group 1: 0.50±0.43 cm2, Group 2: 0.49±0.29 cm2, Group 3: 0,55±0,10 cm2 and Group 4: 0.53±0.30 cm2) of the deviated side of nasal passages (*p*<0.05), but no statistically difference was found in the mean postoperative MCA1 and MCA2 values of the non-deviated side (*p*>0.05). Compared with mean pre and postoperative nasal cavity volumes (Vol1 and Vol2) increased slightly postoperative period but none of these changes had statistical significance (*p*>0.05) (Table 3). 

**Table 3:** Pre and post-operative minimal cross-sectional areas of nasal cavity (MCA1, MCA2) and nasal cavity volumes (VOL1, VOL2) between groups measured by acoustic rhinometry. (♦): Statistically significant (*p*<0.05

**Acoustic ** **rhinometry**	**Deviated side**			**Non-deviated side**		
** MCA1 (cm** ^2^ **) **						
**GROUP** **(n: 10)**	**Pre-op.**	**Post-op.**	***p*** ** value**	**Pre-op.**	**Post-op.**	***p *** **value**
1	0.34±0.28	0.56±0.24	<0.05 (♦)	0.46±0.21	0.50±0.26	>0.05
2	0.33±0.16	0.54±0.29		0.39±0.11	0.41±0.08	
3	0.29±0.20	0.49±0.10		0.41±0.18	0.46±0.26	
4	0.32±0.21	0.51±0.26		0.35±0.15	0.39±0.12	
Control	0.50±0.07			0.52±0.11		
** MCA2 (cm** ^2^ **) **						
**GROUP** **(n: 10)**	**Pre-op.**	**Post-op.**	***p*** ** value**	**Pre-op.**	**Post-op.**	***p*** ** value**
1	0.31±0.35	0.50±0.43	<0.05 (♦)	0.49±0.27	0.48±0.29	>0.05
2	0.29±0.22	0.49±0.29		0.42±0.20	0.43±0.25	
3	0.32±0.30	0.55±0.10		0.39±0.13	0.42±0.34	
4	0.30±0.06	0.53±0.30		0.37±0.15	0.40±0.08	
Control	0.59±0.07			0.63±0.09		
**VOL1 (cm** ^3^ **) **						
**GROUP** **(n: 10)**	**Pre-op.**	**Post-op.**	***p*** ** value**	**Pre-op.**	**Post-op.**	***p*** ** value**
1	1.69±0.79	1.83±0.74	>0.05	1.88±0.39	2.03±0.56	>0.05
2	1.56±0.66	1.74±0.58		1.65±0.40	1.79±0.31	
3	1.73±0.65	1.75±0.40		1.82±0.39	1.91±0.55	
4	1.47±0.58	1.59±0.61		1.64±0.45	1.82±0.51	
Control	1.94±0.20			2.01±0.19		
** VOL2 (cm** ^3^ **) **						
**GROUP** **(n: 10)**	**Pre-op.**	**Post-op.**	***p*** ** value**	**Pre-op.**	**Post-op.**	***p*** ** value**
1	3.85±2.50	4.06±2.54	>0.05	3.93±1.78	4.16±2.12	>0.05
2	3.74±1.19	3.78±2.21		4.05±0.73	4.17±2.14	
3	4.13±1.38	4.29±0.86		4.31±0.93	4.35±2.00	
4	3.88±1.77	4.09±1.46		3.96±1.91	4.18±1.24	
Control	4.17±0.62			4.22±0.62		

## DISCUSSION

Nasal obstruction is a common complaint in the population and may be effected from various physiologic and psychological factors. Most common etiological factor of nasal obstruction is nasal septum deviation. Deviation of septum is diagnosed with complaints of patients, anterior rhinoscopy and nasal endoscopic examination. We studied the same methods used for diagnosis of patients. We obtained detailed anamnesis for all patients and used to evaluate with anterior rhinoscopy and 0° rigid endoscopic nasal examination in preoperative period. Today, the most effective and commonly performed surgery for deviated septum treatment method is septoplasty as we used in our study. Septorhinoplasty is frequently performed which aims to correct functional condition and external deformities of the nose. We used open technique septorhinoplasty which allows best visualization and access. First septal deviations were corrected after hump resection and osteotomy were performed. 

Airway management should not be ignored in septorhinoplasty.^9^ The nasal valve problem was assessed by modified Cottle test and endoscopic nasal examination. Nasal valve has relatively a significant role in the nasal function. Cole *et al.* introduced that very little changes at the nasal valve area could cause a remarkable increase in the nasal resistance.^10^ The nasal valve area is the most restricted section of the upper respiratory tract and it is responsible for about 50% of the airway resistance of the whole respiratory airway.^4^ Several methods including spreader graft, batten grafts and flare sutures are used to support nasal valve area and to avoid nasal obstruction.^5^ We used spreader grafts to correct the nasal valve insufficiency and in addition to contribute to resolve nasal obstruction in patients with nasal valve collapse. In our study, no serious complications (including septum hematoma, septum perforation, wound infections or septum abscesses) were observed.

 Unsuitable indication for nasal surgery was also found as the most important factor for patients’ displeasure.^11^ The postoperative results after nasal surgery are usually based on the subjective feeling by the patient. There is an inadequate data to document whether nasal surgery is efficient in improving symptoms of nasal obstruction. Currently objective and subjective methods are used to evaluate results after nasal surgery. One of the most commonly used objective method is AR and subjective method is NOSE scale as we used in our study.^5^

NOSE scale could be used for comparing disease-specific health status between groups of patients before and after treatment or used to evaluate differences in result when different surgical techniques are performed. Stewart *at al.*^5^ used NOSE scale in 59 patients after septoplasty and Rhee *et al.*^12^ used NOSE scale in 20 patients after nasal valve surgeries to evaluate the quality of life. We observed that there was a very significant improvement in NOSE score at 1 month after nasal surgery and patients also revealed significant decreases in medication use (nasal steroid, antihistaminic and/or oral decongestants) at 1 month after surgeries in our study. 

AR is a simple, non-invasive and inexpensive test. It objectively evaluate and demonstrate the surgical success (septoplasty and other nasal surgeries) by comparing preoperative and postoperative values. It gives cross-sectional areas of the nasal cavity at particular distances from the nostril, in addition to volume measurements. AR may have tolerable accuracy only when done with a decongestant.^13^ In this study, we obtained AR values after decongestion used. According to mean MCA values after operation (MCA1 and MCA2), we found statistically an increase in septal deviation side as compared to before operation values, but we did not observe statistical difference on the side without deviation. These outcomes showed that septum deviation was successfully corrected (according to MCA2 values) and spreader grafts were placed properly (according to MCA1 values). 

Patients’ quality of life was assessed using different scales by many researchers. The most widely used quality of life scales is Healty Measurement Questionnaire (HMQ-2), Short Form 36 (SF-36) and EuroQol (EQ-5D). The significance of disease specific quality of life instrument for nasal obstruction was previously shown.^14^ There are several instruments that are available for use in rhinology, however there are few studies assessing the quality of life on septoplasty and/or septorhinoplasty. Many studies reported high patient satisfaction after septoplasty, but most of them were retrospective.^15^

AR was used to evaluate the nasal obstructions before and after rhinoplasty in 37 patients.^16^ AR could be used to identify the indications for surgical procedure and to assess the postoperative achievement of the surgery in patients with septal deviation. Some authors recommended that AR was a useful method mainly in evaluating anterior nasal space,^17^ and also it was reported that AR was a useful method in the assessment of nasal valve area.^18^ But AR has some restrictions particularly in evaluating the posterior part of the nasal cavity.^19^

NOSE scores and AR values suggested that spreader grafts may be useful in reconstructing the internal nasal valve. A former study of rhinoplasty with spreader grafts and dorsal onlay grafts demonstrated subjective improvement in nasal airflow.^20^ Mamikoglu *et al.* found a poor correlation between clinical findings and AR.^21^ Piril and Tikanto revealed that preoperative AR values had a statistically significant effect in predicting postoperative satisfaction.^22^ Grymer et al. also demonstrated a similar relationship between MCA values and subjective symptoms.^23^

In this study, we tried to assess before and after nasal functions and patient satisfaction that underwent 4 different nasal surgeries by objective and subjective methods. We obtained there was a very significant improvement in NOSE score and also significant increase in MCA values (MCA1 and MCA2) on the septal deviation side of nasal passages at 1 month after nasal surgeries. The success of the surgical procedures can be found in the differences between physician and patient expectations. Expectations of patients from surgery and surgical technique may impact results of surgery. Differences in expectations between patient and physician and may lead to undesirable troubles. For this reason, careful and detailed preoperative patient evaluation is necessary. We observed nasal findings were correlated with NOSE scores and deviation side of AR measurements. According to obtained results, we suggest that NOSE scale and acoustic rhinometry can be used to evaluate the efficiency of functional nasal surgeries.
